# Development of a Prototype for Hepatocellular Carcinoma Classification Based on Morphological Features Automatically Measured in Whole Slide Images

**DOI:** 10.1155/2014/817192

**Published:** 2014-12-30

**Authors:** Yoshiko Yamashita, Tomoharu Kiyuna, Michiie Sakamoto, Akinori Hashiguchi, Masahiro Ishikawa, Yuri Murakami, Masahiro Yamaguchi

**Affiliations:** ^1^NEC Corporation, Tokyo 108-8001, Japan; ^2^Tokyo Institute of Technology, Tokyo 152-8550, Japan; ^3^Keio University, Tokyo 108-8345, Japan; ^4^Saitama Medical University, Saitama 350-1298, Japan

## Introduction

The advent of new digital imaging technologies including high-throughput slide scanners is making a very compelling case as part of the clinical workflow. Tools developed for morphometric image analysis are accelerating the transition of pathology into a more quantitative science. The system for detection of regions suspected to be cancerous in gastric and colorectal tissue is already available. There is a real need for not only cancer detection but also quantification of histological features, because quantitative morphological characteristics can include important diagnostic and prognostic information. If an association between quantitative features and clinical findings is indicated, quantification of morphological features would be extremely useful to select the best treatment. Image measurement technology also has the potential for investigative pathology. We have developed a prototype system for both quantification of morphological features and automated identification of hepatocellular carcinoma (HCC) within whole slide images (WSI) of liver biopsy based on image recognition and measurement techniques. Our system displays quantified cell and tissue features as histogram, bar graph, and heat map on the screen. Displaying all features in such a unified visualization makes it easy to interpret quantitative feature. In this paper, we present a prototype designed specifically for morphological feature visualization in an easy-to-understand manner.

## Method

**Figure 1:**
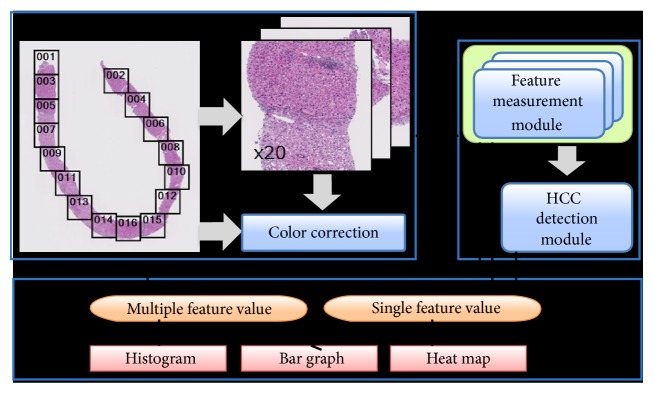
Schematic diagram of prototype system.

Our system automatically analyzes WSI of liver biopsy stained by hematoxylin and eosin (H&E) stain, as shown in Figure 1. Once a WSI is accepted, about 0.9 mm^2^ regions called ROIs (region of interests) are automatically arranged to cover the whole tissue area in a balanced manner. This means that WSI is not cropped into grid tiles. Then, morphological features, such as nuclear and structural atypia level, are extracted and measured from each ROI image in parallel [1, 2]. In addition, the system predicts the HCC [3, 4]. Every feature measurement module outputs multiple and/or single feature values in the same format. In this protocol, our system is designed to add new feature measurement modules easily. And then, the system produces histogram, bar graph, and heat map of all features in the same method.

## Results

**Figure 2:**
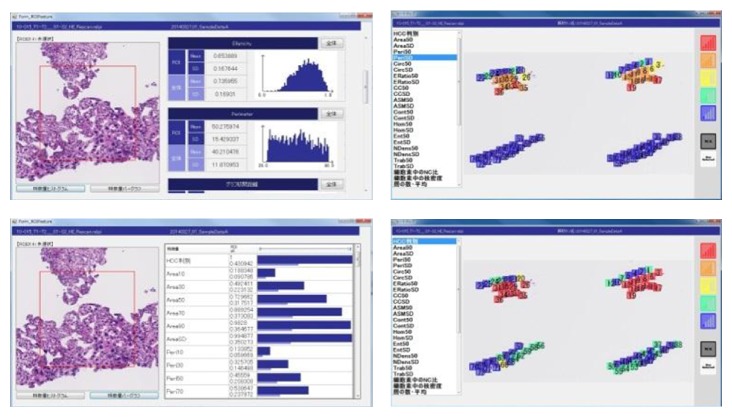
Visualization of morphological features.

The placement of ROIs is in a fine balance, and quantitative morphological features are visually displayed, as shown in Figure 2. The quantification and visualization of histological features are recognized and assessed as informative system.

## Conclusion

Our prototype represents the first step on the path towards a quantitative pathology. The prototype offers a visualization of morphological features. We plan to compute morphological feature values in extensive sets of liver biopsy data using this system and explore the relationship between morphological features and clinical information.
